# The *Salmonella* Deubiquitinase SseL Inhibits Selective Autophagy of Cytosolic Aggregates

**DOI:** 10.1371/journal.ppat.1002743

**Published:** 2012-06-14

**Authors:** Francisco S. Mesquita, Mair Thomas, Martin Sachse, António J. M. Santos, Rita Figueira, David W. Holden

**Affiliations:** 1 Section of Microbiology, Centre for Molecular Microbiology and Infection, Imperial College London, London, United Kingdom; 2 Imagopole, Institut Pasteur, Paris, France; Yale University School of Medicine, United States of America

## Abstract

Cell stress and infection promote the formation of ubiquitinated aggregates in both non-immune and immune cells. These structures are recognised by the autophagy receptor p62/sequestosome 1 and are substrates for selective autophagy. The intracellular growth of *Salmonella enterica* occurs in a membranous compartment, the *Salmonella*-containing vacuole (SCV), and is dependent on effectors translocated to the host cytoplasm by the *Salmonella* pathogenicity island-2 (SPI-2) encoded type III secretion system (T3SS). Here, we show that bacterial replication is accompanied by the formation of ubiquitinated structures in infected cells. Analysis of bacterial strains carrying mutations in genes encoding SPI-2 T3SS effectors revealed that in epithelial cells, formation of these ubiquitinated structures is dependent on SPI-2 T3SS effector translocation, but is counteracted by the SPI-2 T3SS deubiquitinase SseL. In macrophages, both SPI-2 T3SS-dependent aggregates and aggresome-like induced structures (ALIS) are deubiquitinated by SseL. In the absence of SseL activity, ubiquitinated structures are recognized by the autophagy receptor p62, which recruits LC3 and targets them for autophagic degradation. We found that SseL activity lowers autophagic flux and favours intracellular *Salmonella* replication. Our data therefore show that there is a host selective autophagy response to intracellular *Salmonella* infection, which is counteracted by the deubiquitinase SseL.

## Author Summary

Ubiquitination can target substrates to a number of fates, including autophagy, the essential cellular process that allows cells to degrade cytosolic material. Although *Salmonella enterica* resides in a vacuolar compartment during infection, it translocates several virulence proteins into the host cell cytoplasm. We have found that intracellular *Salmonella* induces the formation of ubiquitinated aggregates near the *Salmonella*-containing vacuole and that these aggregates are recognised by the autophagy machinery. *Salmonella* inhibits this response through the action of a translocated enzyme, SseL, which deubiquitinates the aggregates and thereby decreases the recruitment of autophagy markers. We show that SseL alone can deubiquitinate known substrates that are degraded by autophagy, that it reduces autophagy in infected cells and that its activity can increase intracellular *Salmonella* replication. This is a new example of how a bacterium counteracts a cellular defence pathway through the action of a translocated virulence protein.

## Introduction

*Salmonella enterica* is a facultative intracellular pathogen that survives and replicates in a variety of hosts. The virulence traits of *Salmonella* include the *Salmonella* pathogenicity-island (SPI)-1- and -2-encoded type 3 secretion systems (T3SSs), which are important for invasion of host cells [1] and intracellular replication [2], [3], respectively. Intracellular replication occurs in a membrane-enclosed compartment, the *Salmonella*-containing vacuole (SCV), and requires the delivery of an extensive repertoire of effectors to the host cytoplasm by the SPI-2 T3SS [4].

Ubiquitination is an essential process in eukaryotic cells that regulates protein degradation, localization and function. The ability to manipulate the ubiquitin system is a feature common to many intracellular pathogens, including *Salmonella*, which delivers several effectors to the cell cytosol that interfere with the host cell ubiquitination [5], [6]. Absence of the SPI-2 T3SS-delivered deubiquitinase SseL leads to an accumulation of ubiquitinated proteins within infected cells and attenuates *Salmonella* virulence in mice [7].

Intracellular bacteria generate a diverse array of substrates that are ubiquitinated during infection. These include vacuolar membrane remnants produced after intracellular lysis of bacterial vacuoles [8], bacterial cell wall products [9] and protein aggregates or aggresome-like induced structures (ALIS) [10]–[12]. In addition, bacterial LPS, cell stress and toll-like receptor (TLR) signalling can trigger the formation of ALIS in several cell types, including macrophages [13], [14]. ALIS and other ubiquitinated protein aggregates are targeted by ubiquitin binding proteins such as p62/sequestosome 1 (p62 hereafter), which can lead to receptor-mediated selective macroautophagy (hereafter referred to as selective autophagy).

In response to this cellular defence pathway, bacteria have evolved different ways to interfere with selective autophagy. *Listeria* ActA recruits the Arp2/3 complex and Ena/VASP to the surface of cytosolic bacteria to prevent recognition by ubiquitin and p62 [15]. Likewise, *Shigella* camouflages its surface through binding of the T3SS effector protein IcsB to the bacterial surface protein IcsA/VirG, thereby preventing the recognition of VirG by the autophagy-related protein, Atg5 [16], and avoiding recruitment of ubiquitin and p62 [17].

The ubiquitination and selective autophagy of cytosolic *Salmonella* has been studied extensively by others [18]–[20]. In this work, we demonstrate that *Salmonella* within vacuoles induces a cellular response leading to the formation of SPI-2 T3SS-dependent ubiquitinated aggregates and ALIS during infection. We show that a SPI-2 T3SS effector, SseL, deubiquitinates these aggregates and prevents the recruitment of the autophagy markers p62 and LC3. SseL deubiquitinase activity leads to a reduction in autophagic flux during infection and promotes intracellular bacterial replication.

## Results

### Ubiquitinated structures accumulate near *Salmonella*-containing vacuoles

In epithelial cells, replicating *Salmonella* form a cluster of SCVs (referred to as a microcolony) close to the microtubule organizing centre and Golgi apparatus [21]. Bacterial replication is accompanied by dramatic reorganization of late endosomal compartments [22], and condensation of actin [23], microtubules [24] and intermediate filament networks [25], in close proximity to SCVs. These processes could perturb cellular homeostasis and cause cell stress. Since cell stress often leads to the appearance of inclusions containing ubiquitinated proteins [11], [13] confocal microscopy was used to analyse the localization of mono- and poly-ubiquitinated proteins in relation to bacterial microcolonies in HeLa cells infected with *Salmonella enterica* serovar Typhimurium (*S.* Typhimurium) strains.

**Figure 1:**
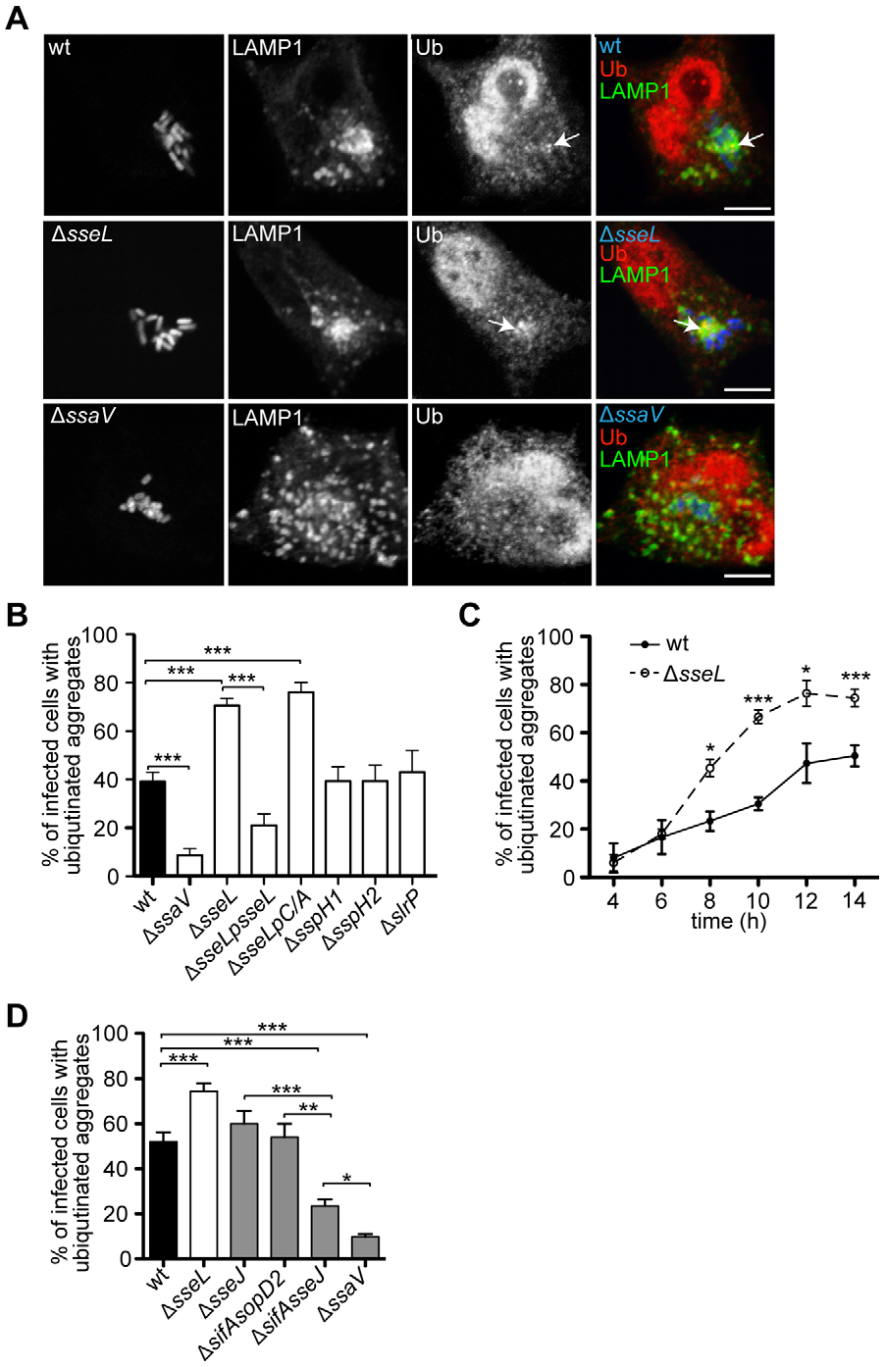
Ubiquitinated aggregates accumulate near SCVs in HeLa cells. (A) Single confocal sections of HeLa cells infected with GFP-expressing *S.* Typhimurium strains (blue) for 10 h, fixed and immunolabelled for LAMP1 (green) and ubiquitin (Ub, red) (false coloured, scale bars, 5 µm). The far right panels show merged images of LAMP1, ubiquitin and *Salmonella*. Arrows indicate SCV-associated ubiquitinated aggregates. (B) Quantification of the percentage of infected cells containing SCV-associated ubiquitinated aggregates. Cells were processed as in (A) and analysed by fluorescence microscopy. (C) Kinetics of the formation of SCV-associated ubiquitinated aggregates. Cells were infected with GFP-expressing *S.* Typhimurium strains for the indicated amount of time, and processed as in (A). (D) Quantification of the percentage of infected cells with SCV-associated ubiquitinated aggregates in HeLa cells infected with *S.* Typhimurium strains for 14 h. Cells were processed as in (A) and analysed by fluorescence microscopy. For all quantifications, a minimum of 50 infected cells were counted per experiment and values are the mean ± SEM from at least 3 independent experiments. * p<0.05; ** p<0.01; *** p<0.001.

At 10 h after invasion, in addition to characteristic labelling of the nucleus, punctate ubiquitin labelling was observed close to bacterial microcolonies in approximately 40% of cells infected with wild-type bacteria (Fig. 1A and 1B). In contrast, less than 10% of cells infected with an *ssaV* null mutant (Δ*ssaV*) strain (lacking a functional SPI-2 T3SS) had such structures (Fig. 1A and 1B), even after infection for 14 h, when the numbers of intracellular *ΔssaV* bacteria were similar to those of the wild-type strain at 10 h (Fig. 1D). This suggested that translocation of SPI-2 T3SS effectors is required for the formation of the majority of these ubiquitinated aggregates. Several SPI-2 T3SS effectors directly interfere with ubiquitin pathways by acting as E3 ligases (SspH1, SspH2 and SlrP) [26], [27] or as a deubiquitinase (SseL) [6]. SCV-associated ubiquitinated aggregates in cells infected with strains lacking the E3 ligases were similar to those in cells infected with wild-type bacteria (Fig. 1B) and were also present following infection with a triple mutant strain lacking all three E3 ligases (Fig. S1). In contrast, 75% of cells infected with *ΔsseL* mutant bacteria showed striking SCV-associated ubiquitin labelling (Fig. 1A and 1B), indicating that the presence of SseL results in the loss of ubiquitinated structures near SCVs. These results suggest that SPI-2 T3SS effector translocation induces the accumulation of aggregates close to SCVs that are ubiquitinated by unknown E3 ligase(s) and are deubiquitinated by SseL.

### SseL deubiquitinase activity inhibits the accumulation of ubiquitinated aggregates

To establish that the deubiquitinase activity of SseL was required to reduce the frequency of SCV-associated ubiquitinated aggregates, HeLa cells were infected with a *Salmonella ΔsseL* mutant strain complemented with plasmid-expressed epitope-tagged versions of SseL: SseL or SseLC262A (SseLC/A), a mutant that lacks a catalytic cysteine required for its activity [7]. Complementation with wild-type SseL dramatically reduced the frequency of cells containing ubiquitinated structures, whereas translocated SseLC/A had no noticeable effect, despite extensive co-localization with ubiquitin (Fig. 1B and S2A). Ubiquitin structures within clusters of at least five bacteria were detected from approximately 6 h after bacterial invasion, coinciding with the onset of SPI-2 T3SS effector translocation [28], [29] (Fig. 1C); infections with bacteria lacking SseL resulted in a consistent increase in their frequency from 8 h onwards (Fig. 1C). Deletion of *sseL* had no major effect on the number of ubiquitinated cytosolic bacteria occurring at early time-points after invasion (Fig. S2C), indicating that the accumulation of ubiquitin near SCVs is distinct from the ubiquitination of the small proportion of cytosolic *Salmonella*.

### Endosomal remodelling is required for the accumulation of ubiquitinated aggregates

Colocalization between SCV-associated ubiquitin labelling and the lysosomal membrane glycoprotein LAMP1 (Fig. 1A) suggested that manipulation of host vesicular trafficking pathways might be involved in the accumulation of ubiquitin. *Salmonella* SPI-2 T3SS effectors, including SifA, induce and regulate extensive tubular networks called *Salmonella*-induced filaments (sifs) that are enriched in late endosomal markers such as LAMP1 [30] and cause the collapse of endosomal compartments around SCVs [31]. To investigate whether there was a link between the accumulation of the ubiquitinated aggregates and these effects we analysed HeLa cells infected with additional *S.* Typhimurium mutant strains.

Deletion of *sifA* alone results in instability of the SCV membrane and bacterial escape [32], [33], therefore we analysed HeLa cells infected with double mutant strains containing mutations in either *sifA* and *sseJ* or *sifA* and *sopD2*, as these strains have increased membrane stability [34], [35]. Because the *sifA*, *sseJ* double mutant strain has a reduced replication rate [36], cells were examined 14 h after invasion, when it was possible to compare cells with similar numbers of bacteria as cells infected with wild-type bacteria at 10 h after invasion (Fig. 1D). At this time point only 25% of the cells infected with *sifA*, *sseJ* double mutant strain contained SCV-associated ubiquitinated aggregates (Fig. 1D). However, cells infected with a *sifA*, *sopD2* double mutant or *sseJ* mutant strain contained the same frequency of ubiquitinated structures observed with wild-type *Salmonella* (Fig. 1D).

*Salmonella* also induces extensive rearrangement of actin and microtubules around SCVs [23], [24]. To determine whether these processes are required for the formation of ubiquitinated aggregates, HeLa cells were infected with single mutant strains lacking SseG, which is required for formation of bundles of microtubules [37] or SteC, which causes assembly of an SCV-associated F-actin meshwork [38]. Both these strains induced ubiquitinated aggregates to similar levels as seen in cells infected with wild-type bacteria (data not shown). Hence, accumulation of these aggregates is not linked to positioning of SCVs or actin polymerization, but is at least in part due to the combined actions of SifA and SseJ.

### Ubiquitin accumulates in dense cytosolic aggregates and forms K63-linked chains

**Figure 2:**
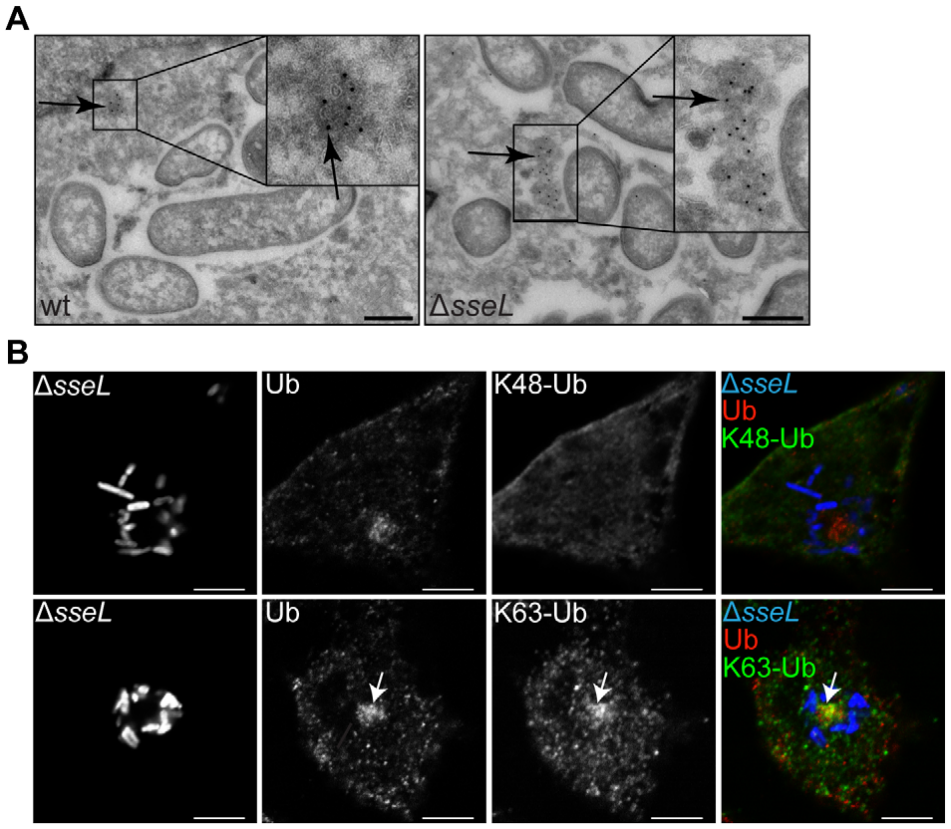
Ubiquitin accumulates in dense cytosolic aggregates and forms K63-linked chains. (A) Immunoelectron microscopy of HeLa cells infected with *S.* Typhimurium strains for 12 h (ubiquitin - 10 nm gold particles; scale bars, 0.5 µm). Arrows indicate ubiquitin accumulations near bacteria within vacuoles. (B) Single confocal sections of HeLa cells infected with GFP-expressing Δ*sseL* mutant bacteria (blue) for 10 h and immunolabelled for ubiquitin (all chain types - Ub, red) and lysine-48-linked (K48-Ub, green) or lysine-63-linked (K63-Ub, green) ubiquitin chains (false coloured, scale bars, 5 µm). Arrows indicate SCV-associated ubiquitin and K63-Ub labelling.

The ultrastructure of these ubiquitinated aggregates was examined by transmission immunoelectron microscopy (IEM). Ubiquitin labelling was observed in electron-dense structures in both wild-type and *ΔsseL* infected cells (Fig. 2A). In agreement with our observations by confocal microscopy, ubiquitin labelling was found more frequently in *ΔsseL* than wild-type infected cells and was found in very close proximity to SCVs. No labelling was found within the luminal space or on bacteria by IEM. Further immunofluorescence analysis of these structures using antibodies recognizing either lysine 48-linked (K48) or lysine 63-linked (K63) ubiquitin chains revealed that K63 but not K48 ubiquitin chains were present in these structures (Fig. 2B), consistent with the known preference of SseL for K63 chains [7]. However, co-localization between the FK2 antibody (which labels all ubiquitin chains) and the K63 antibody was incomplete; therefore it is possible that other ubiquitin topologies might also be present.

### p62 and LC3 localize to SCV-associated ubiquitinated aggregates

Autophagy is required for the clearance of various types of ubiquitinated aggregates induced following bacterial infection [39]. This clearance can involve p62, which interacts with both ubiquitin chains and LC3 to promote the autophagic degradation of protein inclusions [40].

**Figure 3:**
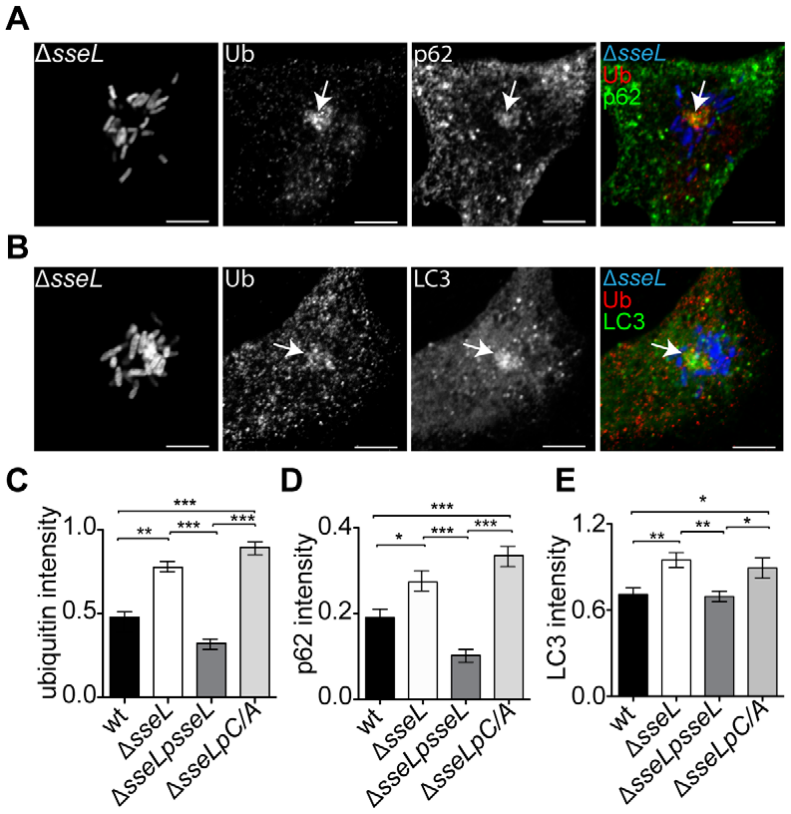
SseL DUB activity reduces the recruitment of ubiquitin and autophagic markers to *Salmonella* microcolonies. Single confocal sections of HeLa cells infected with GFP-expressing Δ*sseL* mutant bacteria (blue) for 12 h and immunolabelled for ubiquitin (Ub, red) and (A) p62 (green) or (B) LC3 (green) (false coloured, scale bars, 5 µm). The far right panels show merged images of p62 or LC3, ubiquitin and *Salmonella*. Arrows indicate SCV-associated ubiquitin labelling with p62 or LC3. (C–E). Quantification of the relative fluorescence intensity of (C) ubiquitin, (D) p62 and (E) LC3 per microcolony (see Materials and Methods). Cells were processed as in (A) and a minimum of 30 microcolonies analysed for each bacterial infection per experiment. Results from representative experiments showing the relative mean fluorescence intensity ± SEM are shown. Similar results were obtained in at least 3 independent experiments. * p<0.05; ** p<0.01; *** p<0.001.

We analysed the distribution of p62 and LC3 in HeLa cells infected with wild-type or *ΔsseL* mutant bacteria. Dense punctate labelling of both p62 and LC3 was detected close to SCVs, and this partly co-localized with ubiquitin (Fig. 3A and 3B). To examine the influence of SseL on these proteins, confocal microscopy was used to measure the relative fluorescence intensity of ubiquitin, p62 and LC3 labelling in SCV-associated structures. The absence of SseL significantly increased the intensity of ubiquitin, p62 and LC3 labelling, compared to cells infected with wild-type bacteria (Fig. 3C–E). This was complemented by a plasmid expressing wild-type SseL, but not by expression of SseLC/A (Fig. 3C–E).

The presence of ubiquitin and p62 within the same aggregates was confirmed by IEM analysis of infected HeLa cells, which showed dense structures in the vicinity of SCVs co-labelled with p62 and ubiquitin (Fig. S3A). Likewise, a HeLa cell line stably expressing GFP-LC3 also contained ubiquitin and GFP-LC3 positive structures close to SCVs (Fig. S3B). Together, these results demonstrate that the deubiquitinase activity of SseL reduces the presence of ubiquitin, p62 and LC3 in SCV-associated aggregates.

### SCV-associated aggregates are regulated by autophagy

**Figure 4:**
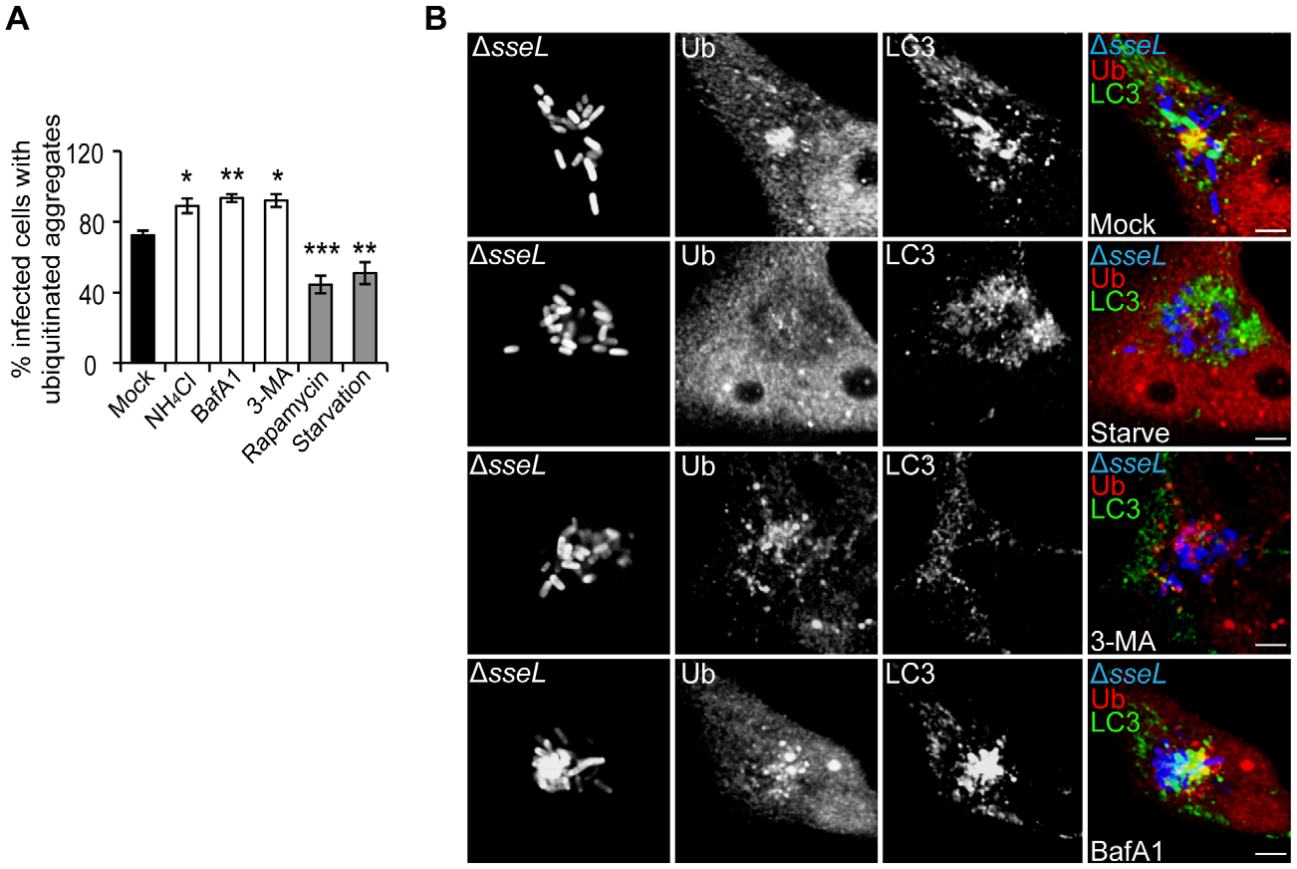
SCV-associated ubiquitinated aggregates are autophagy substrates. (A) Quantification of the percentage of infected cells with SCV-associated ubiquitinated aggregates. HeLa cells were infected with *ΔsseL* mutant bacteria for 12 h. At 8 h post-infection, cells were subjected to the indicated treatments for 4 h before fixation and immunolabelling. A minimum of 50 cells were counted per condition in each experiment and values are the mean ± SEM of at least 3 independent experiments. *p* values are relative to the mean value of mock-treated cells. * p<0.05; ** p<0.01; *** p<0.001. (B) Single confocal sections of HeLa cells infected with GFP-expressing *ΔsseL* mutant bacteria (blue), treated and processed as in (A) and analysed by fluorescence microscopy for ubiquitin (Ub, red) and LC3 (green) (scale bars, 5 µm). The far right panels show merged images of LC3, ubiquitin and *Salmonella*.

To examine the role of autophagy in the regulation of these structures, HeLa cells infected with *ΔsseL* mutant bacteria were subjected to conditions that either inhibit or stimulate autophagy and examined for the presence of SCV-associated ubiquitinated aggregates (Fig. 4). 3-methyladenine (3-MA), ammonium chloride (NH_4_Cl) or bafilomycin A1 (BafA1) were used to inhibit autophagy at early (3-MA) and late (NH_4_Cl and BafA1) stages of the pathway; starvation and rapamycin were used to stimulate autophagy. To avoid any interference with the maturation of the SCV and early targeting of cytosolic bacteria, infected cells were maintained in normal medium until 8 h after invasion. Cells were then exposed to autophagy-altering conditions for 4 h before processing for confocal microscopy. The frequency of ubiquitinated aggregates was enhanced significantly upon inhibition of autophagosome formation (3-MA) or autophagic degradation (BafA1 and NH_4_Cl), whereas stimulation of autophagy by starvation or rapamycin strongly reduced their presence (Fig. 4A and 4B). The alterations in autophagic flux were confirmed by an increased presence of LC3-positive compartments around SCVs in starved cells (Fig. 4B); LC3-positive compartments were almost undetectable in cells exposed to 3-MA despite the increase in ubiquitin labelling (Fig. 4B). BafA1, which inhibits degradation of lysosomal contents, enhanced both ubiquitin and LC3 labelling of these compartments (Fig. 4B). These results show that the ubiquitinated aggregates induced by SPI-2 T3SS effectors are substrates of the autophagy pathway.

### SseL deubiquitinates ALIS

**Figure 5:**
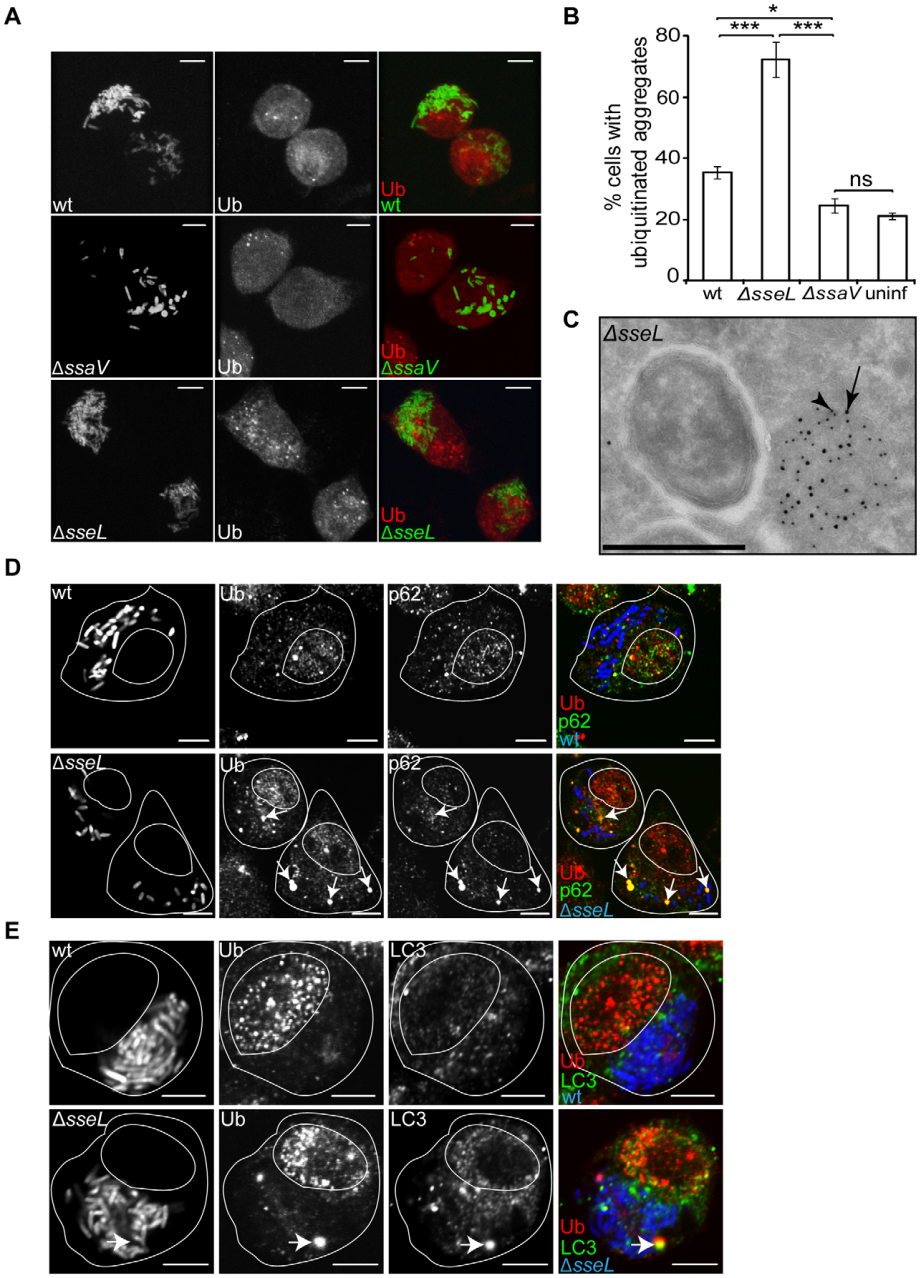
SseL reduces the accumulation of ubiquitinated aggregates and ALIS in macrophages. (A) Single confocal sections of RAW264.7 macrophages infected with GFP-expressing strains of *S.* Typhimurium (green) for 10 h and immunolabelled for ubiquitin (Ub, red) (scale bars, 5 µm). (B) Quantification of infected macrophages containing ubiquitinated aggregates 10 h after bacterial uptake. Cells were processed as in (A) and analysed by fluorescence microscopy. A minimum of 50 cells were counted for each bacterial infection per experiment and values are the mean ± SEM of at least 3 independent experiments. * p<0.05; *** p<0.001. Uninfected cells (uninf) were from the same wells as infected and therefore were exposed to extracellular bacteria. (C) Immunoelectron microscopy of RAW264.7 macrophages infected with the *ΔsseL* mutant bacteria for 12 h. Arrows and arrowheads indicate ubiquitin (15 nm gold particles) and p62 (10 nm gold particles), respectively (scale bar, 0.5 µm). (D and E) Single confocal sections of RAW264.7 macrophages infected with GFP-expressing strains of *S.* Typhimurium (blue) and immunolabelled for ubiquitin (Ub, red) and (D) p62 (green) or (E) LC3 (green). The far right panels show merged images of p62 or LC3, ubiquitin and *Salmonella*. Arrows indicate Arrows indicate SCV-associated ubiquitin labelling with p62 or LC3 (scale bars, 5 µm). Cell outlines and nuclei are delineated by white lines.

Stimulation of macrophages and dendritic cells with LPS induces the formation of cytosolic ubiquitinated aggregates that are subject to autophagy. These are referred to as aggresome-like induced structures (ALIS) [11], [13], [40]. We showed previously that lysates from mouse macrophages infected with *ΔsseL* mutant bacteria are enriched in ubiquitinated proteins [7]. Immunofluorescence and IEM analysis of infected RAW264.7 macrophages revealed the presence of small ubiquitin puncta similar to those seen in epithelial cells, as well as larger, more spherical aggregates that were particularly evident in cells infected with the *ΔsseL* mutant strain (Fig. 5). In RAW264.7 macrophages, these larger structures were up to approximately 0.5 µm in diameter, similar to the size of ALIS [14] (Fig. 5C), and they co-labelled with p62 (Fig. 5C and 5D) and LC3 (Fig. 5E). Fluorescence microscopy revealed that HA-tagged SseLC/A co-localized to approximately 50% of ALIS in infected RAW264.7 macrophages (data not shown) and the effector was also visualised on cytosolic structures co-labelled with ubiquitin by IEM (Fig. S2B).

As there was a continuum of different sized puncta in RAW264.7 macrophages, the percentage of infected cells with any ubiquitin-positive aggregates was quantified. Approximately 35% of macrophages infected with the wild-type strain contained ALIS and other aggregates 10 h after bacterial uptake (Fig. 5A and 5B). 24% of cells infected with *ΔssaV* mutant strain also contained these structures, indicating that the majority of aggregates in macrophages are induced independently of SPI-2 T3SS function. Indeed, a similar proportion of uninfected cells that had been exposed to bacteria contained ubiquitinated aggregates (Fig. 5B). Since it is known that *E. coli* and *S.* Typhimurium LPS induce ALIS in macrophages [13], [14], LPS is likely to account for the SPI-2-independent induction of ubiquitinated aggregates.

**Figure 6:**
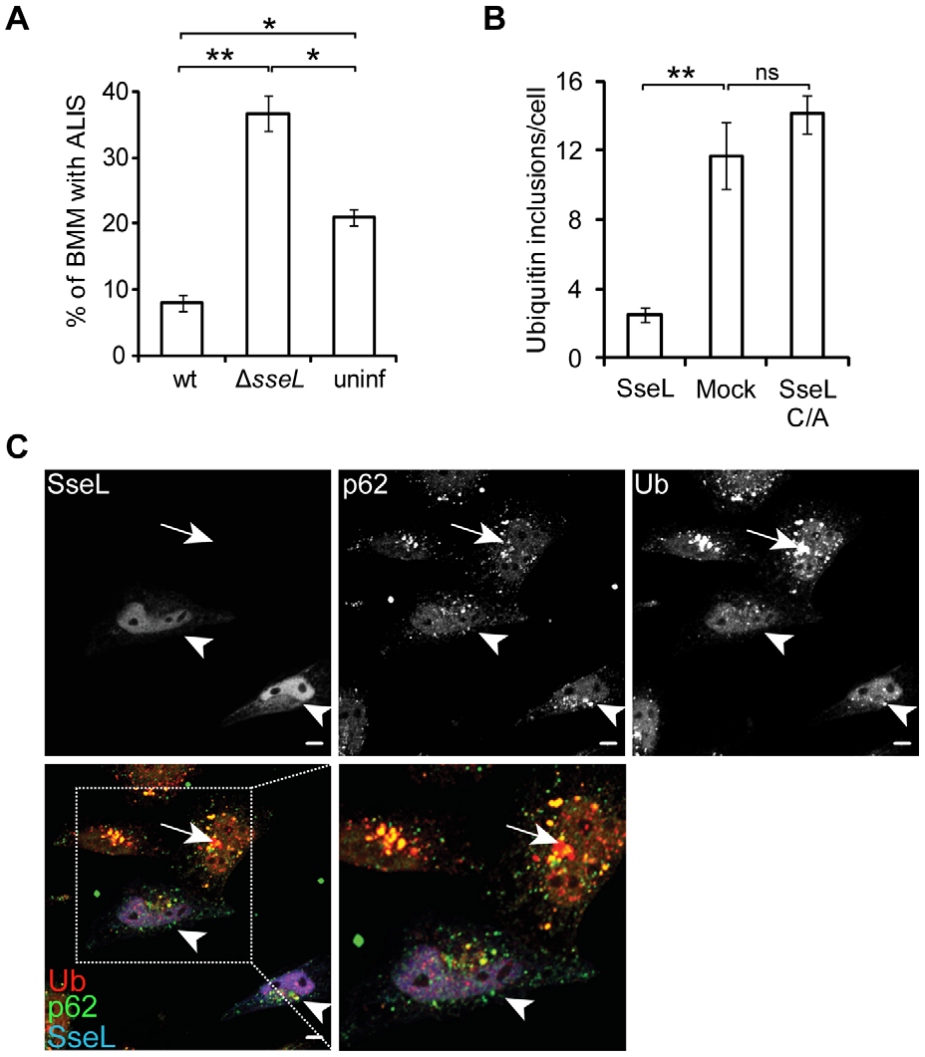
SseL deubiquitinates *Salmonella*- and puromycin-induced ALIS. (A) Quantification of large (>2 µm), ubiquitinated aggresome-like induced structures (ALIS) in macrophages. Primary bone marrow-derived macrophages (BMM) were infected with the indicated GFP-expressing *S.* Typhimurium strains for 10 h, immunolabelled for ubiquitin and analysed by confocal microscopy. A minimum of 50 cells were counted for each bacterial infection per experiment and values are the mean ± SEM of 3 independent experiments. Uninfected cells (uninf) were from the same wells as infected and therefore were exposed to extracellular bacteria. * p<0.05; ** p<0.01. (B) Quantification of the number of puromycin-induced ubiquitin inclusions in cells. HeLa cells were transfected with a vector expressing myc-SseL or myc-SseLC/A for 16 h followed by treatment with puromycin (5 µg/ml) for 4 h and immunolabelled with anti-myc, anti-p62 and anti-ubiquitin. 50 individual cells were counted per condition in each experiment. Values are the means ± SEM of at least 3 independent experiments. ** p<0.01. (C) Single confocal sections of HeLa cells transfected with a vector expressing myc-SseL for 16 h followed by treatment with puromycin (5 µg/ml) for 4 h and immunolabelled with anti-myc (blue), anti-p62 (green) and anti-ubiquitin (Ub, red) (scale bars, 5 µm). The lower panels show a merged image of p62, ubiquitin and myc-SseL. Arrows indicate ubiquitin and p62 aggregates present in untransfected cells and arrowheads indicate cells expressing myc-SseL.

Approximately 70% of RAW264.7 macrophages infected with *ΔsseL* mutant bacteria contained ubiquitinated aggregates (Fig. 5B). SseL-dependent deubiquitination of aggregates was also observed in infected mouse bone marrow-derived primary macrophages (Fig. S4). These cells contained large ALIS that were readily distinguishable from SCV-associated aggregates. Their quantitation revealed that wild-type *Salmonella*-infected cells had fewer ALIS than uninfected cells in the same well, whereas cells infected with *ΔsseL* mutant bacteria had a far greater number (Fig. 6A). Uninfected cells not exposed to extracellular bacteria did not contain any ALIS (Fig. S5).

To establish whether SseL was sufficient to deubiquitinate independently-generated ALIS, we transiently transfected HeLa cells with vectors encoding myc-tagged SseL or myc-tagged SseLC/A. LPS does not induce ALIS in HeLa cells, but puromycin (which causes premature chain termination during translation) leads to the accumulation of defective ribosomal products (DRiPs) that are ubiquitinated and incorporated into inclusion bodies. These aggregates also contain p62 and are indistinguishable from LPS-induced ALIS [11], [14], [40]. HeLa cells were either mock transfected or transfected with a vector expressing myc-tagged SseL or SseLC/A, then treated with puromycin for 4 h and analysed for ALIS. There were approximately 5-fold fewer ALIS in cells expressing SseL compared to mock-transfected or SseLC/A-expressing cells (Fig. 6B and 6C). Collectively, these experiments show that SseL is sufficient to deubiquitinate proteins in *Salmonella-* or puromycin-induced ALIS.

### SseL interacts with p62 and autophagic substrates

**Figure 7:**
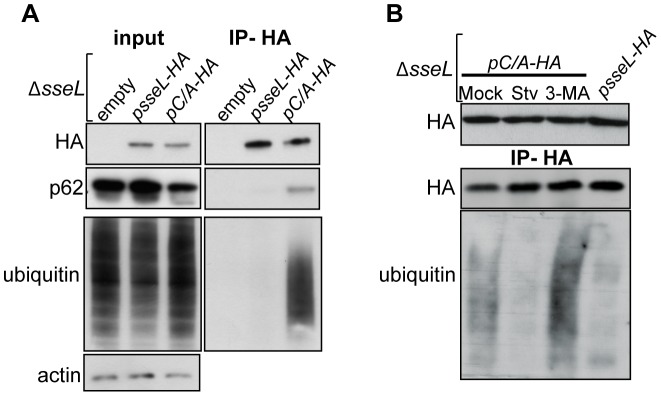
SseL interacts with p62 and autophagic substrates. (A and B) Co-immunoprecipitation from RAW264.7 macrophages infected with *ΔsseL* mutant bacteria expressing SseL-HA or SseLC/A-HA for 10 h. (A) Infectedcell lysates and immunoprecipated fractions were probed with anti-HA, anti-ubiquitin and anti-p62 antibodies. (B) At 7 h post-infection, infected cells were subjected to mock, 3-MA or starvation (Stv) treatments for 3 h before harvesting and processing as described in (A). Cell lysates and immunoprecipiations were probed with anti-HA and anti-ubiquitin antibodies.

The catalytically inactive SseL mutant (SseLC/A) binds stably to ubiquitinated substrates and allows their immunoprecipitation [7]. To determine if SseL can co-immunoprecipitate autophagic substrates, macrophages were infected with the *sseL* deletion mutant expressing either wild-type SseL-HA or SseLC/A-HA from a plasmid, and after 10 h of infection, lysates were immunoprecipitated with an anti-HA antibody (Fig. 7A). Immunoblotting revealed that p62 was specifically co-immunoprecipitated by SseLC/A-HA (Fig. 7A). A similar result was obtained using infected HeLa cells (data not shown). This shows that SseL and p62 might bind to the same ubiquitinated substrates. Furthermore, since p62 was immunoprecipitated by SseLC/A but not wild-type SseL (Fig. 7A), it is likely that this interaction occurs indirectly through the ubiquitinated substrates. Next we examined the effects of autophagy on the accumulation of SseL-interacting ubiquitinated substrates. Infected macrophages were subjected to 3 h of 3-MA or starvation treatment to inhibit or stimulate autophagic flux respectively before cell lysis. 3-MA markedly increased the levels of ubiquitinated proteins interacting with SseLC/A (Fig. 7B). In contrast, starvation conditions virtually eliminated these substrates (Fig. 7B). Therefore, SseL deubiquitinates substrates targeted by p62 and destined for autophagic degradation.

### SseL reduces autophagic flux and influences bacterial replication in macrophages

**Figure 8:**
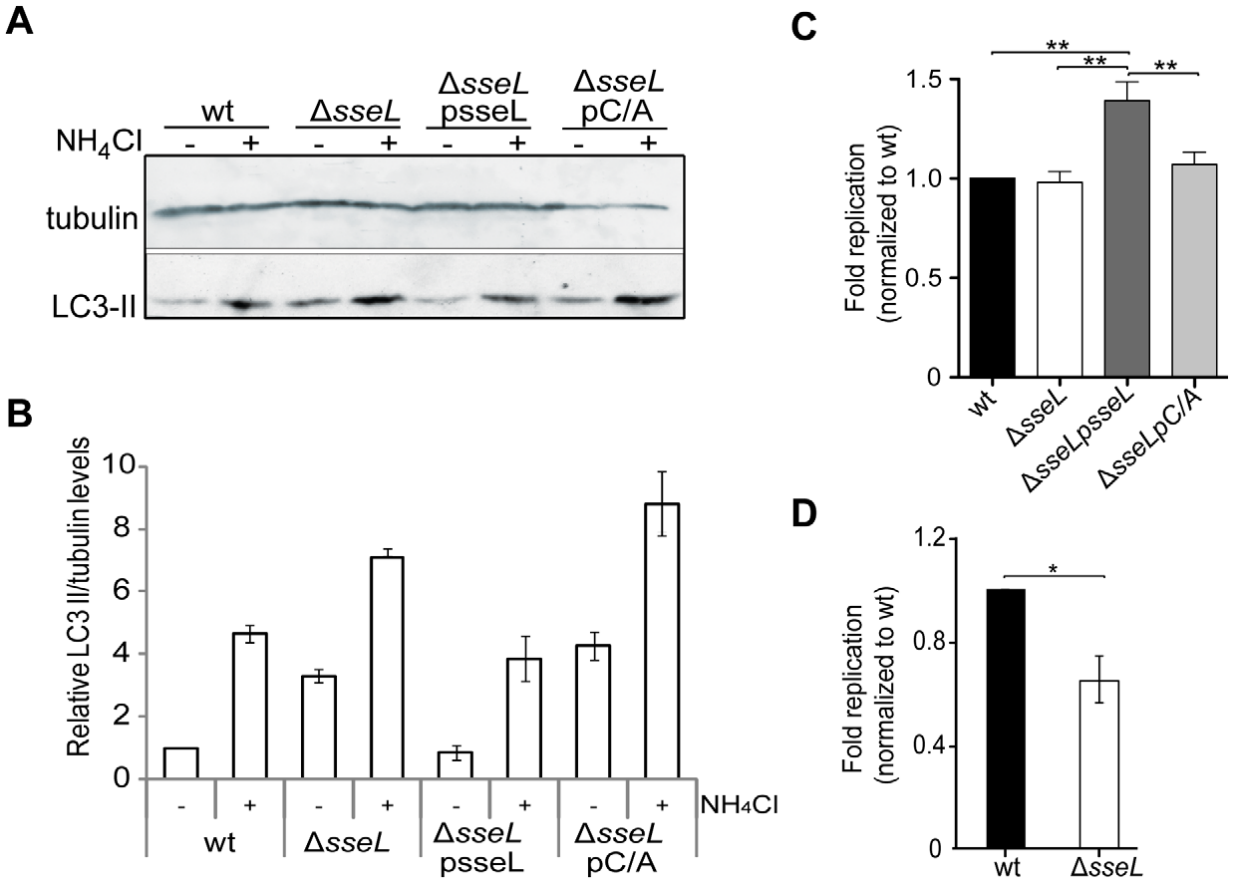
SseL reduces autophagic flux and can promote bacterial replication in macrophages. (A) Lipidated LC3B (LC3-II) levels in RAW264.7 macrophages infected with the indicated strains of *S.* Typhimurium for 12 h. Cells were left untreated (−) or subjected to 10 mM NH_4_Cl treatment (+) for 2 h before harvesting. (B) Quantification of relative LC3 II levels in infected cells. Denistometry of bands was analysed using Image J software. The ratio of LC3-II/tubulin signal intensity was normalized to 1 for wild-type untreated infections. The graph shows the mean relative LC3 II/tubulin ratios ± SEM for 3 independent experiments. (C) RAW264.7 or (D) murine bone marrow-derived primary macrophages were infected with the indicated strains of *S.* Typhimurium for (C) 10 h or (D) 16 h and bacterial replication was monitored using flow cytometry. Results are represented as fold replication normalized to wt; values are the means ± SEM of at least 3 independent experiments. * p<0.05; ** p<0.01.

Accumulation of protein aggregates has been shown to induce autophagy in models of Huntington's disease [41]. In addition, LPS-induced ALIS formation increases autophagic flux in macrophages [11], [42]. Since SseL deubiquitinates ALIS, we analysed autophagic flux by determining LC3-II levels in infected macrophages. When LC3 is conjugated with phosphatidylethanolamine (LC3-II) it localizes to autophagosomal membranes [43] and the amount of LC3-II correlates with the number of autophagosomes present in cells. Exposure of cells to NH_4_Cl to inhibit lysosomal degradation allows differentiation between an increase in autophagic flux and impaired autophagic degradation [43], [44]. Cells infected with *sseL* mutant bacteria had increased levels of LC3-II compared to wild-type infected cells and this was complemented when bacteria expressed wild-type SseL but not SseLC/A (Fig. 8A and B). Following exposure to NH_4_Cl, the levels of LC3-II were still higher in the absence of SseL (Fig. 8A and B), indicating that the increase in LC3-II observed in cells infected with bacteria lacking SseL did not result from a block in autophagosomal degradation. Therefore, the absence of SseL deubiquitinase activity stimulates autophagic flux, presumably by increasing the accumulation of ubiquitinated aggregates.

Previous studies have demonstrated that SseL contributes to the virulence of *S.* Typhimurium in mice, but failed to show an intracellular growth defect of *sseL* mutant strains [7], [45]. In view of its effect on ALIS, we reassessed the contribution of SseL to intracellular bacterial replication using fluorescence dilution, a technique which enables direct measurement of bacterial replication at the single cell level [3]. The replication of wild-type and *ΔsseL* mutant bacteria was indistinguishable 10 h after uptake into RAW264.7 cells (Fig. 8C). The mean replication of both strains was approximately 40-fold at 10 h, as calculated by fluorescence dilution [3]. However, *ΔsseL* mutant bacteria complemented with a plasmid overexpressing SseL underwent a ∼35% increase in bacterial replication. This increase was not observed for isogenic bacteria expressing the catalytically inactive SseLC/A (Fig. 8C). RAW264.7 cells are relatively permissive for replication of *S.* Typhimurium [3]. To examine the replication in a more physiological and restrictive environment, primary macrophages were also used to compare replication of the *sseL* mutant (Fig. 8D). Mouse bone marrow-derived macrophages were infected for 16 h and bacterial replication was determined by fluorescence dilution. The replication of *sseL* mutant *Salmonella* at 16 h (10.5-fold) was significantly lower than that of wild-type bacteria (17-fold) (Fig. 8D), indicating that SseL contributes to replication in this more restrictive cellular environment.

## Discussion

We have shown that intravacuolar *Salmonella* induces the formation of ubiquitinated aggregates containing autophagic markers in both epithelial cells and macrophages. In epithelial cells, rather diffuse ubiquitin puncta were found close to SCVs, and these partially co-localized with LAMP1 and electron-dense material. These structures were SPI-2 T3SS-dependent and required the combined activities of SifA and SseJ, effectors that regulate vacuolar membrane dynamics and which perturb the endosomal network [35]. Infected macrophages contained similar structures and also dense, more spherical aggregates that resemble ALIS. Although such ALIS have not been characterized previously in *Salmonella*-infected cells, they are similar to those induced in macrophages following bacterial LPS stimulation [13], [14]. The aggregates found in *Salmonella*-infected macrophages resembled LPS-induced ALIS in their size, shape and recruitment of p62 and LC3 [14], suggesting that LPS signalling through TLR4 might trigger their formation. Remarkably, both aggregates and ALIS in epithelial cells and macrophages are targets for the SPI-2 T3SS effector, SseL, which deubiquitinates as yet unidentified p62-bound proteins and reduces the recruitment of autophagic machinery.

It was reported recently that knockout of the deubiquitinase AMSH (associated molecule with the SH3 domain of STAM) in mice caused accumulation of ubiquitinated inclusions containing p62 in neurons [46]. In addition, LPS-induced autophagic signalling can be reduced by the deubiquitinase A20 which deubiquitinates K63-linked chains from Beclin-1 [47]. However, it is not known whether A20 or AMSH act directly on substrates of autophagy or whether their effects are indirect. Therefore, to our knowledge SseL is the first deubiquitinase to be described that targets material destined for autophagic degradation, and this represents a new mechanism by which *Salmonella* counteracts a cellular response induced by an innate immune signalling pathway. It is likely that a proportion of the ubiquitinated aggregates found in macrophages form in response to LPS-TLR4 signalling, as *S.* Typhimurium LPS stimulates ALIS formation in RAW 264.7 macrophages [13] and LPS-induced ALIS in macrophages have been shown to require TLR4 signalling [14]. However other bacterial products may also be involved; *Listeria monocytogenes* culture broth, zymosan beads as well as heat treatment and cell stress have been shown to induce ALIS formation in several cell types [11], [13]. The presence of ubiquitinated aggregates in *Salmonella*-infected macrophages might also be inhibited by other effectors that interfere with TLR-dependent immune signalling, such as the phosphothreonine lyase SpvC, which acts on MAP kinases downstream of MyD88 [48].

Ubiquitinated aggregates that are targeted by selective autophagy have been described in cells exposed to other bacterial pathogens, but these have mainly been cytosolic or extracellular bacteria [8], [9], [49], [50]. However, expression of the *Legionella pneumophila* Dot/Icm type IV secretion system in macrophages was shown to reduce ALIS formation during infection [12]. Although a *Legionella* deubiquitinase has not yet been identified, these observations together with our results suggest that such an activity might exist. Indeed it is possible that deubiquitinases of other pathogens might target ubiquitinated aggregates in a similar way, and their activity could mask the presence of these aggregates.

An inability to degrade misfolded or aggregated proteins can lead to cell death, as seen in neurodegenerative disorders [51]. Autophagy ameliorates the cellular stress induced by the presence of protein aggregates [52]. Therefore it is possible that the reduction in autophagic flux mediated by SseL accounts for its ability to induce delayed cytotoxicity in macrophages [7]. While the possible functions of ALIS remain unclear, they serve to sequester cytosolic proteins under a variety of stress conditions and are suggested to be involved in host immune defence [14], [53], [54]. The ubiquitinated proteins in ALIS are considered as a source of antigenic peptides for presentation on MHC class I molecules in dendritic cells [14], [53], [54]; therefore the deubiquitination of aggregates by SseL might regulate antigen presentation in infected macrophages. It will be important to determine the fate of proteins contained within *Salmonella*-induced aggregates following deubiquitination by SseL. As ubiquitin, p62 and LC3 are currently the only markers of these structures, we are unable to assess whether they persist in cells following their deubiquitination. The presence of non-ubiquitinated protein inclusions may also adversely affect the cell and impede bacterial replication. The identity of the substrates within aggregates that are targeted for deubiquitination by SseL remains elusive. The ability of exogenously expressed SseL to target puromycin-induced ALIS suggests that it may be a promiscuous deubiquitinase that can target a number of substrates and that its activity is regulated by its localization to the SCV during infection.

It is known that the activity of SseL contributes to *Salmonella* growth *in vivo* [7]. In the present study we found that SseL also contributes to intracellular replication in macrophages. In the permissive RAW264.7 macrophage cell line, no differences in replication were observed between wild-type bacteria and the *ΔsseL* mutant, but an increase in replication occurred upon over-expression of active SseL. However, in the more restrictive bone-marrow derived macrophages, which better represent the cells that *Salmonella* encounters *in vivo*, replication was reduced in the absence of SseL.

Recent work has proposed that SseL binds to oxysterol-binding protein (OSBP) [55], and interferes with lipid metabolism, leading to the clearance of lipid droplets, which otherwise accumulate during infection of mice gallbladder epithelial cells [56]. Lipid droplet metabolism has been associated with autophagy [57]; therefore it would be interesting to study the possible relationship between the inhibition of selective autophagy by SseL and its interference with lipid droplet metabolism. Understanding how SseL interferes with selective autophagy and contributes to bacterial replication and cytotoxicity will be helped by the identification of molecules present in the ubiquitinated aggregates and the ubiquitin-conjugated proteins that are targeted by SseL.

Many intracellular bacteria produce effectors that are known to interfere with ubiquitin-related processes, suggesting that modulating this pathway is important for bacterial growth and survival inside cells. During and following cell invasion, the ubiquitination machinery is used by *Salmonella* SPI-1 T3SS effectors to regulate both their degradation and cellular localization [58], [59]. Our work has shown that ubiquitinated aggregates are formed at late time-points in infected cells, in response to both SPI-2 T3SS-dependent rearrangement of endocytic compartments and to SPI-2 T3SS-independent signals. We found that *Salmonella* uses the SPI-2 T3SS effector SseL to deubiquitinate proteins in these aggregates, thereby impeding their autophagic degradation. The possible links between this interference of autophagy and SseL-associated cytotoxicity and replication will be the subject of future research.

## Materials and Methods

### Bacterial strains, plasmids and culture conditions

Standard microbial techniques were used for construction of strains and plasmids. Detailed information on the *S.* Typhimurium strains and plasmids used in this study are provided in Tables S1 and S2 respectively [60], [61]. Strains harbouring the pDiGc plasmid were cultured in MgM-MES minimal medium supplemented with 0.2% L-arabinose at 37°C with aeration [3]. All other strains were grown in Luria Bertani (LB) medium, at 37°C with aeration. When appropriate, cultures were supplemented with kanamycin (50 µg/ml) and carbenicillin (50 µg/ml). The chromosomal deletions of *avrA*, *sspH1* and *sspH2* in *S.* Typhimurium were performed by one-step gene-disruption method [62]. *sspH2* and *slrP* mutations were transduced into *ΔsspH1* and *ΔsspH1ΔsspH2* mutant strains of *S.* Typhimurium respectively by P22 phage transduction.

### Cell culture and DNA transfection

HeLa (cloneHtA1) and RAW264.7 cells were obtained from the European Collection of Animal and Cell Cultures, Salisbury, UK. Cells were grown in Dulbecco's modified Eagle medium (PAA laboratories) supplemented with 10% foetal calf serum (PAA laboratories) at 37°C in 5*%* CO_2_. Primary bone marrow-derived murine macrophages (BMM) were obtained from C57BL/6 mice (Charles River) extracted from tibia and femur [63] and grown as described [3]. HeLa cells were transfected using Lipofectamine 2000 (Invitrogen) transfection reagent according to the manufacturer's instructions.

### Bacterial infections

HeLa and RAW264.7 macrophage infections were performed as previously described [32] except for infections carried out with strains harbouring pDiGc, which were grown overnight in MgM-MES supplemented with 0.2% L-arabinose [3]. Primary BMM were infected as described [3]. For all experiments, coverslips from 1 h post-inoculation were labelled to verify that there were similar levels of infection between strains and conditions. In BMM, approximately 50% of cells were infected.

### Antibodies

For confocal microscopy, FK2 mouse anti-mono and polyubiquitinated proteins (ENZO) was used at 1∶10,000; CSA-1 goat anti-*Salmonella* (Kirkegaard and Perry Laboratories) at 1∶400; 931A rabbit anti-LAMP1 [64] at 1∶1000; Clone Apu3 rabbit anti-K63 ubiquitin chains (Millipore) at 1∶1000; Clone Apu2 rabbit anti-K48 ubiquitin chains (Millipore) at 1∶1000; rabbit anti-p62 (ENZO) at 1∶1000; rabbit anti-LC3B (MBL) at 1∶1000; 3H603 rat anti-myc (Santa Cruz) at 1∶200; rat anti-HA (Roche) at 1∶200. Secondary antibodies were obtained from Invitrogen: Alexa 488-, Alexa 555- and Alexa 633-conjugated donkey anti-goat, anti-rabbit, anti-mouse, or anti-rat antibodies were used for immunofluorescence at a dilution of 1∶400. For immunoblotting, antibodies were used at following dilutions: P4D1 mouse anti-ubiquitinated proteins (Santa Cruz) 1∶1000; HA11 mouse anti-HA (Covance) 1∶5000; rabbit anti-actin (Sigma) 1∶5000; rabbit anti-p62 at 1∶1000 (MBL); anti-mouse (IgG) and anti-rabbit (IgG) horseradish peroxidase secondary antibodies (GE Healthcare) at 1∶10,000. For immunoelectron microscopy the following antibodies and reagents were used: rabbit anti-GFP (Rockland) at 1∶100; mouse anti-rabbit (Dako) at 1∶300; rabbit anti-p62 (ENZO) at 1∶30; and Protein A gold from the Cell Microscopy Center, UMC Utrecht, The Netherlands.

### Reagents and drug treatments

All drugs were prepared in stock solutions and stored as indicated by the manufacturer. 3-Methyladenine (3-MA) (Sigma) was used at 10 mM [65]; bafilomycin A1 (BafA1) (Sigma) at 200 nM [40]; rapamycin (Sigma) at 0.2 mg/ml [66]; puromycin (Sigma) at 5 µg/ml [40]. For starvation treatments cells were incubated in Hank's buffer salt solution (HBSS). NH_4_Cl was used at 10 mM.

### Confocal immunofluorescence microscopy

For confocal microscopy, samples were fixed in 4% formaldehyde and permeabilized in 0.2% Triton X-100 for 5 min. All antibodies were diluted to the appropriate concentration in PBS containing 10% horse serum. The coverslips were washed twice in PBS, incubated with primary antibodies for 1 h, washed 3 times in PBS and incubated with secondary antibodies for 30 min. Coverslips were washed and mounted onto glass slides using Mowiol mounting medium. Cells were observed with a confocal laser scanning microscope (Zeiss Axiovert LSM510) or an epifluorescence microscope (BX50 Olympus Optical). Images were processed using Zeiss LSM510 image software and Adobe Photoshop software. In all figures apart from S2A, single confocal sections are shown. In Fig. S2A a projection of stacked confocal sections is shown.

### Puromycin-induced inclusion assays

HeLa cells were transfected with 500 ng of pRK5::myc-SseL [67], pRK5::myc-SseLC/A or empty pRK5 for 16 h and cells were subjected to puromycin treatment for additional 4 h until processing for confocal microscopy. The number of ubiquitin inclusions per cell was quantified for at least 50 cells for each sample.

### Immunofluorescence quantifications

To quantify the percentage of cells displaying ubiquitinated structures around bacterial microcolonies (a cluster of at least five SCVs with overlapping fluorescence signals) HeLa cells, RAW264.7 macrophages or BMM were infected with strains of *S.* Typhimurium and immunolabelled for mono and poly-ubiquitinated proteins. Detection of the bacteria was done by using GFP-expressing strains of *S.* Typhimurium [68] or by immunolabelling with anti-*Salmonella* antibodies. Cells were scored positive if the bacterial fluorescence signal overlapped ubiquitin labelling. At least 50 infected cells were counted for each sample in at least three independent experiments. Only cells containing a minimum of five bacteria and a maximum of 20 bacteria were counted. For quantification of the numbers of ubiquitinated bacteria, at least 100 bacterial cells were counted for each sample and analysed for pixel to pixel colocalization between ubiquitin and GFP-expressing bacteria. The percentage of cells containing at least one singular ubiquitinated bacterium was obtained by counting 50 infected cells for each sample for each time-point. All quantifications were done in a blind manner for at least 3 independent experiments.

### Quantitative analysis of confocal projections

For the quantification of ubiquitin, p62 or LC3 on bacterial microcolonies, HeLa cells were infected with strains *S.* Typhimurium immunolabelled for mono and polyubiquitinated proteins, p62, LC3 and *Salmonella*. 3D confocal stacks were acquired for each sample using a slice increment of 0.4 µm. These stacks were then analysed as 3D images using the Volocity software. A protocol in the Volocity software was created and identical settings used for analysis of all experiments. Intracellular bacteria were selected according to an arbitrary threshold of fluorescence intensity of anti-*Salmonella* labelling for all images. Manual manipulation ensured that only cells with at least 5 bacteria clustered in a perinuclear position were selected. All intracellular bacteria were then grouped as one individual object containing the volume of cellular cytoplasm. The mean values of fluorescence intensity of ubiquitin, p62 or LC3 labelling present within individual microcolonies were quantified and divided by the mean values of fluorescence intensity of bacterial labelling. Data were expressed as the ratio of mean fluorescence of ubiquitin, p62 or LC3 to the fluorescence of bacteria. At least 30 individual microcolonies were quantified for each sample per individual experiment in a minimum of 3 independent experiments (minimum of 90 microcolonies in total).

### Immunoelectron microscopy (IEM)

For IEM of HeLa cells or RAW264.7 macrophages, cells were infected with different strains of *S.* Typhimurium for 12 h, then fixed, immunolabelled and embedded as previously described [9]. Single labeling to reveal FK2 antibody and sequential double labeling were performed according to the protein A gold method [69].

### Immunoblot analysis of LC3B

For immunoblot analysis of the levels of LC3B, RAW264.7 macrophages (2×10^6^ cells) infected with strains of *S.* Typhimurium were left untreated or incubated with 10 mM NH_4_Cl for 2 h before harvesting. To ensure equivalent bacterial loads, coverslips were examined at 1 h post-uptake and for all experiments, approximately 70% of RAW264.7 macrophages were infected by all strains. 12 h after bacterial uptake cells were washed and harvested in ice cold PBS and centrifuged at 14,000× *g* for 2 min. Cells were lysed in TBS (20 mM Tris-Cl, pH 7.5 150 mM NaCl) containing 1% Triton X-100, 2.5 mM MgCl_2_, 1 mM PMSF and 1× protease inhibitor cocktail (Roche) and lysates were centrifuged at 14,000× *g* at 4°C for 10 min. Supernatants were resuspended in sample buffer (0.25 mM Tris-Cl pH 6.8, 10% SDS, 50% glycerol, 5% β-mercaptoethanol) and subjected to SDS-PAGE followed by immunoblot analysis. Immunoblots were developed using HRP-conjugated secondary antibodies and visualised using ECL (GE healthcare). Densitometry was performed on film by high-resolution scanning and analysis using ImageJ software (NIH).

### Co-immunoprecipitations

For HA immunoprecipitations, RAW264.7 macrophages (6×10^6^ cells) infected with strains of *S.* Typhimurium for 12 h were washed, harvested in cold PBS and centrifuged at 200× *g* at 4°C. Cells were lysed in RIPA buffer (Sigma) containing 1 mM PMSF and 1× protease inhibitor cocktail (Roche) and left on ice for 30 min. The lysates were centrifuged at 14,000× *g* for 10 min and the supernatants were pre-cleared with protein G-immobilized beads (Pierce). After a brief centrifugation supernatants were incubated overnight at 4°C with 30 µl of protein G-immobilized beads conjugated to 1 µg of HA11 mouse anti-HA antibody overnight at 4°C. Samples were then centrifuged for 30 sec at 14,000× *g*, and washed twice in lysis buffer. Immunoprecipitated proteins were eluted from beads in sample buffer and subjected to SDS-PAGE followed by immunoblot analysis.

### Replication assays

Infections were carried out with strains harbouring pDiGc as described. Briefly, bacteria were grown overnight in MgM-MES supplemented with 0.2% L-arabinose prior to infection. At appropriate time points after infection, intracellular bacteria released from macrophages were analysed by flow cytometry on a two-laser, four colour FACS Calibur™ flow cytometer. Data were analysed with FlowJo software version 8.1.1 (TreeStar). Fold replication values from 2 h to the indicated time points were calculated and normalised to wt. Results represent the means ± SEM of at least 3 independent experiments.

### Statistical analysis

All statistical analyses were performed with Prism 5 software (GraphPad) using one-way_ANOVA_ with Dunnett *post hoc* analyses to compare different means in relation to a control sample, with Newman-Keuls *post hoc* analyses for pairwise comparisons of more than 2 different means or using two-tailed un-paired Student's t-test for comparison of means between 2 samples. For *p* values<0.05, the differences between samples were considered as statistically significant. In all figures, * *p*<0.05; ** *p*<0.01; *** *p*<0.001.

## Supporting Information

Figure S1 **Formation of SCV-associated ubiquitinated aggregates is independent of the SPI-2 T3SS-delivered E3 ubiquitin ligases.** (A) Single confocal sections of HeLa cells infected with the indicated strains of *S.* Typhimurium for 10 h and immunolabelled for ubiquitin (Ub, red) and *Salmonella* (green). The far right panels show merged images of ubiquitin and *Salmonella*. Cell outlines and nuclei are delineated by white lines (scale bars, 5 µm). Arrows indicate SCV-associated ubiquitin accumulations.
(TIF)

Figure S2 **SseL deubiquitinates SCV-associated ubiquitinated aggregates and ALIS but does not affect ubiquitination of cytosolic bacteria.** (A) Projection of stacked confocal sections of HeLa cells infected with the indicated strains of *S.* Typhimurium for 10 h and immunolabelled for ubiquitin (Ub, red), HA (green) and *Salmonella* (blue). (B) Immunoelectron microscopy of RAW264.7 macrophages infected with *ΔsseL* mutant bacteria expressing SseLC/A-HA. Arrow indicates ubiquitin - 15 nm gold particles; arrowhead indicates HA – 10 nm gold particles (scale bar, 0.5 µm). (C) Quantification of the percentage of intracellular bacteria that are ubiquitinated over a time-course of infection. Immunofluorescence quantification of HeLa cells infected with GFP-expressing *S.* Typhimurium strains for the indicated times, fixed and immunolabelled for ubiquitin. 100 bacterial cells were counted for each strain at each time-point. All values are the means ± SEM of 3 independent experiments.
(TIF)

Figure S3 **Electron dense SCV-associated ubiquitinated aggregates contain p62 and GFP-LC3 in infected HeLa cells.** (A) Immunoelectron microscopy of HeLa cells infected with *ΔsseL* mutant bacteria for 12 h (arrowheads indicate ubiquitin - 15 nm gold particles; arrows indicate p62 – 10 nm gold particles; scale bar, 0.5 µm). (B) Immunoelectron microscopy of HeLa cells stably expressing GFP-LC3 infected with *ΔsseL* mutant bacteria for 12 h (arrowheads indicate ubiquitin - 15 nm gold particles; arrows indicate GFP-LC3 – 10 nm gold particles; scale bar, 0.5 µm).
(TIF)

Figure S4 **SseL inhibits accumulation of SCV-associated ubiquitinated aggregates in infected primary macrophages.** (A) Representative single confocal sections of primary bone marrow-derived macrophages (BMM) infected with GFP-expressing strains of *S.* Typhimurium (green) for 16 h and immunolabelled for ubiquitin (Ub, red). Arrows indicate ubiquitin aggregates. Cell outlines and nuclei are delineated by white lines (scale bars, 5 µm). (B) Immunoblot analysis of ubiquitinated proteins from lysates of BMM infected with the indicated strains of *S.* Typhimurium for 16 h or 20 h using anti-ubiquitin and anti-actin antibodies. (C) Quantification of SCV-associated ubiquitinated aggregates at 16 h after bacterial uptake in BMM. Cells were processed as in (A) and analysed by fluorescence microscopy. A minimum of 50 cells were counted for each sample and values are the mean ± SEM of at least 4 experiments independent experiments. ** p<0.01.
(TIF)

Figure S5 **Confocal images of ubiquitin levels in primary macrophages.** (A) Representative single confocal sections of murine primary bone marrow-derived macrophages infected with the indicated strains of GFP-expressing *Salmonella* (green) for 10 h or uninfected (and unexposed to bacterial products). Cells were fixed and labelled for ubiquitin (Ub, red) and analysed by confocal microscopy (scale bars, 20 µm).
(TIF)

Table S1 **Bacterial strains used in this work.**
(DOCX)

Table S2 **Plasmids used in this work.**
(DOCX)
