# Supplementary figures and images for: The Salmonella Deubiquitinase SseL Inhibits Selective Autophagy of Cytosolic Aggregates
Source: PLoS Pathog. 2012 Jun 14;8(6):e1002743. doi: 10.1371/journal.ppat.1002743 (PMC3375275; doi:10.1371/journal.ppat.1002743)

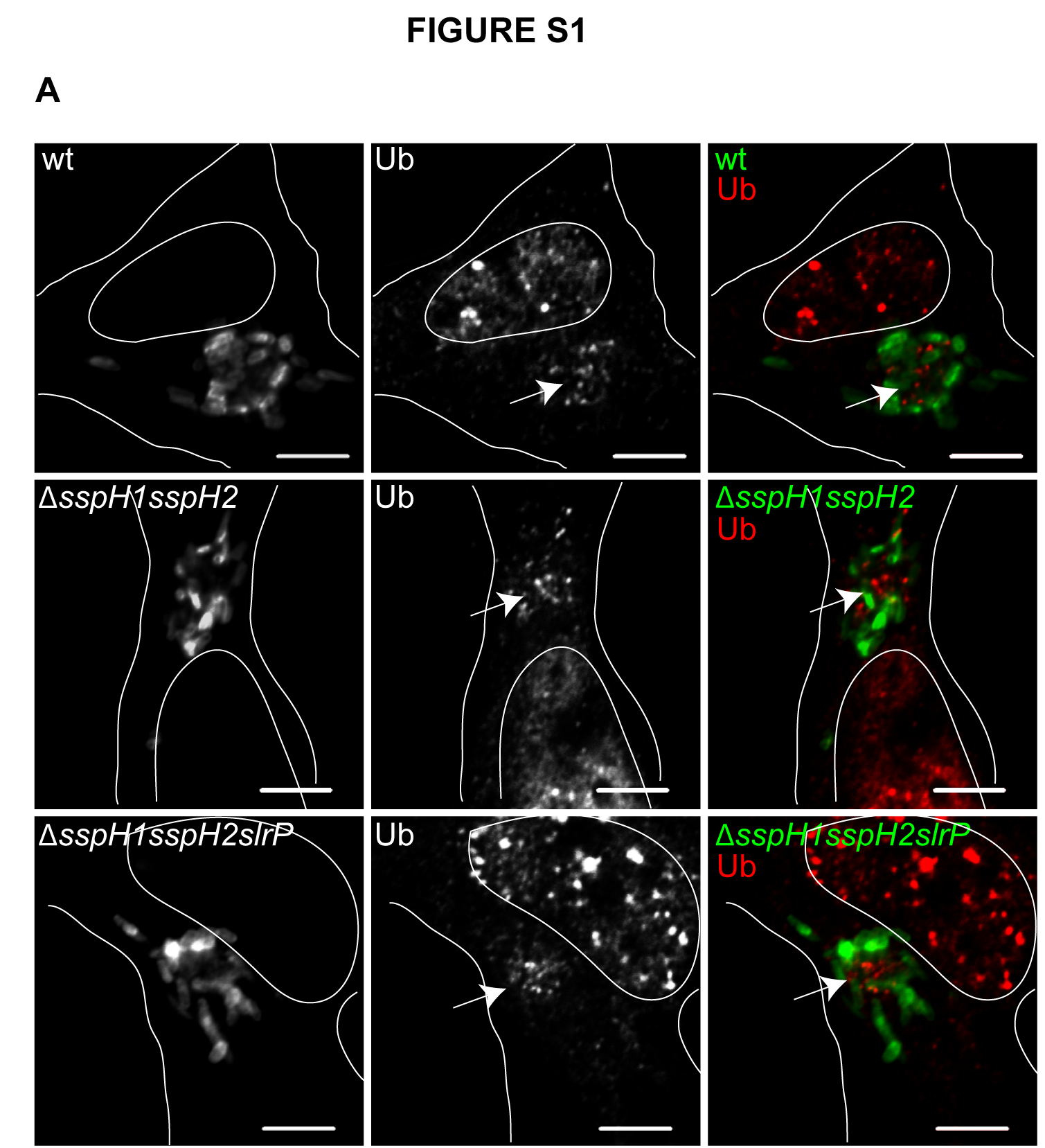

Supplement: Figure S1 — Formation of SCV-associated ubiquitinated aggregates is independent of the SPI-2 T3SS-delivered E3 ubiquitin ligases. (A) Single confocal sections of HeLa cells infected with the indicated strains of S. Typhimurium for 10 h and immunolabelled for ubiquitin (Ub, red) and Salmonella (green). The far right panels show merged images of ubiquitin and Salmonella. Cell outlines and nuclei are delineated by white lines (scale bars, 5 µm). Arrows indicate SCV-associated ubiquitin accumulations. (TIF) [file ppat.1002743.s001.tif]

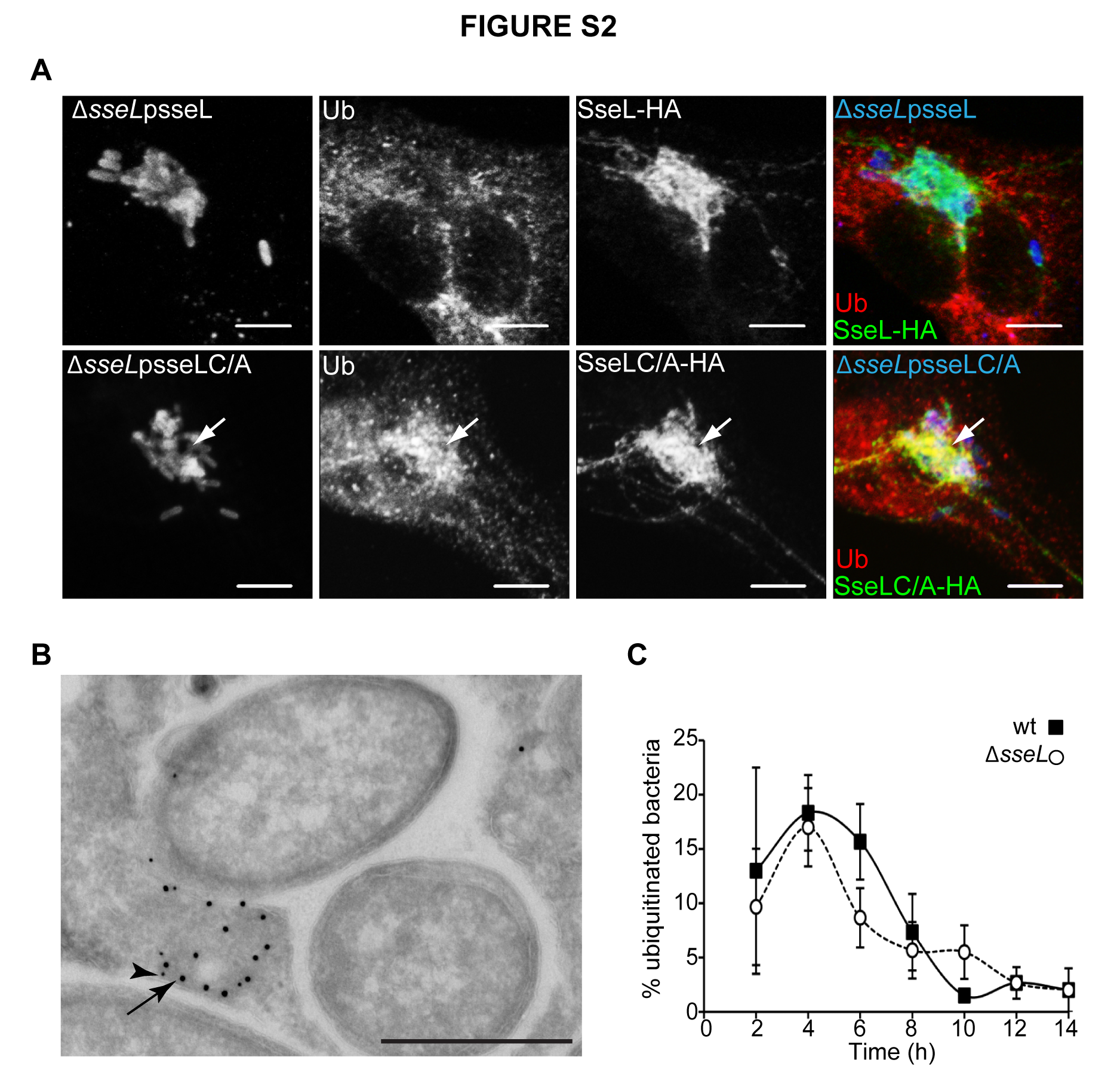

Supplement: Figure S2 — SseL deubiquitinates SCV-associated ubiquitinated aggregates and ALIS but does not affect ubiquitination of cytosolic bacteria. (A) Projection of stacked confocal sections of HeLa cells infected with the indicated strains of S. Typhimurium for 10 h and immunolabelled for ubiquitin (Ub, red), HA (green) and Salmonella (blue). (B) Immunoelectron microscopy of RAW264.7 macrophages infected with ΔsseL mutant bacteria expressing SseLC/A-HA. Arrow indicates ubiquitin - 15 nm gold particles; arrowhead indicates HA – 10 nm gold particles (scale bar, 0.5 µm). (C) Quantification of the percentage of intracellular bacteria that are ubiquitinated over a time-course of infection. Immunofluorescence quantification of HeLa cells infected with GFP-expressing S. Typhimurium strains for the indicated times, fixed and immunolabelled for ubiquitin. 100 bacterial cells were counted for each strain at each time-point. All values are the means ± SEM of 3 independent experiments. (TIF) [file ppat.1002743.s002.tif]

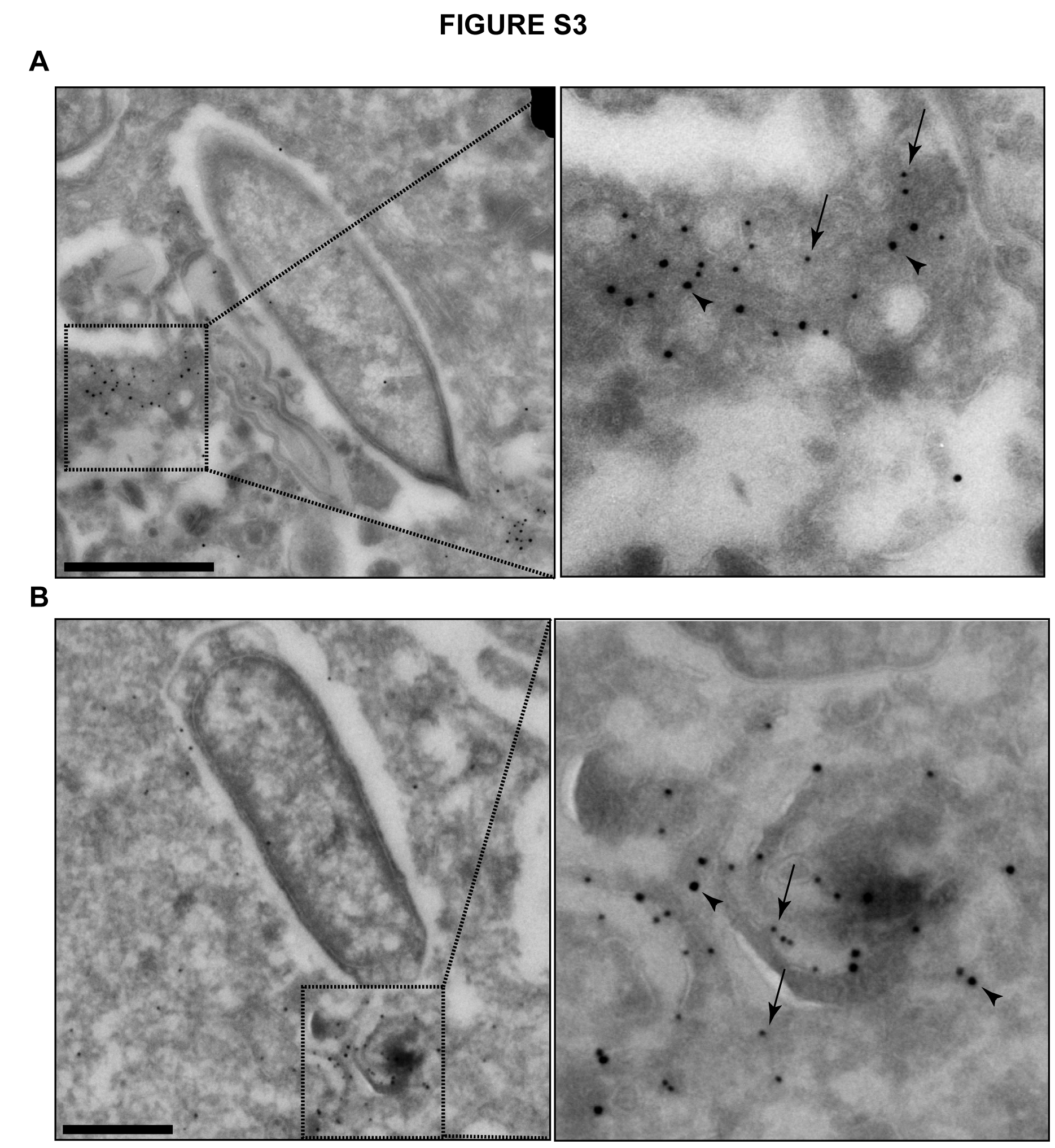

Supplement: Figure S3 — Electron dense SCV-associated ubiquitinated aggregates contain p62 and GFP-LC3 in infected HeLa cells. (A) Immunoelectron microscopy of HeLa cells infected with ΔsseL mutant bacteria for 12 h (arrowheads indicate ubiquitin - 15 nm gold particles; arrows indicate p62 – 10 nm gold particles; scale bar, 0.5 µm). (B) Immunoelectron microscopy of HeLa cells stably expressing GFP-LC3 infected with ΔsseL mutant bacteria for 12 h (arrowheads indicate ubiquitin - 15 nm gold particles; arrows indicate GFP-LC3 – 10 nm gold particles; scale bar, 0.5 µm). (TIF) [file ppat.1002743.s003.tif]

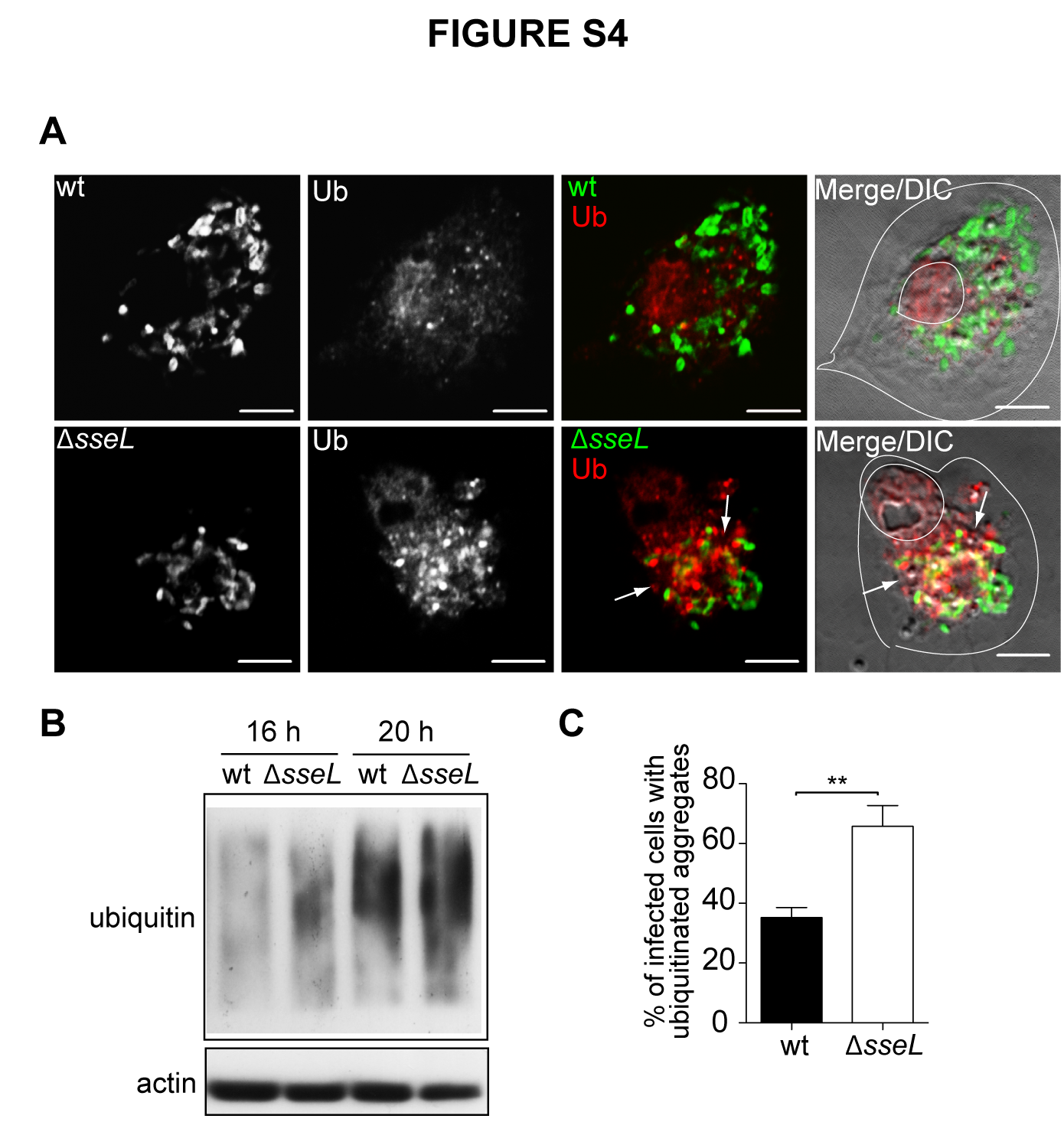

Supplement: Figure S4 — SseL inhibits accumulation of SCV-associated ubiquitinated aggregates in infected primary macrophages. (A) Representative single confocal sections of primary bone marrow-derived macrophages (BMM) infected with GFP-expressing strains of S. Typhimurium (green) for 16 h and immunolabelled for ubiquitin (Ub, red). Arrows indicate ubiquitin aggregates. Cell outlines and nuclei are delineated by white lines (scale bars, 5 µm). (B) Immunoblot analysis of ubiquitinated proteins from lysates of BMM infected with the indicated strains of S. Typhimurium for 16 h or 20 h using anti-ubiquitin and anti-actin antibodies. (C) Quantification of SCV-associated ubiquitinated aggregates at 16 h after bacterial uptake in BMM. Cells were processed as in (A) and analysed by fluorescence microscopy. A minimum of 50 cells were counted for each sample and values are the mean ± SEM of at least 4 experiments independent experiments. ** p<0.01. (TIF) [file ppat.1002743.s004.tif]

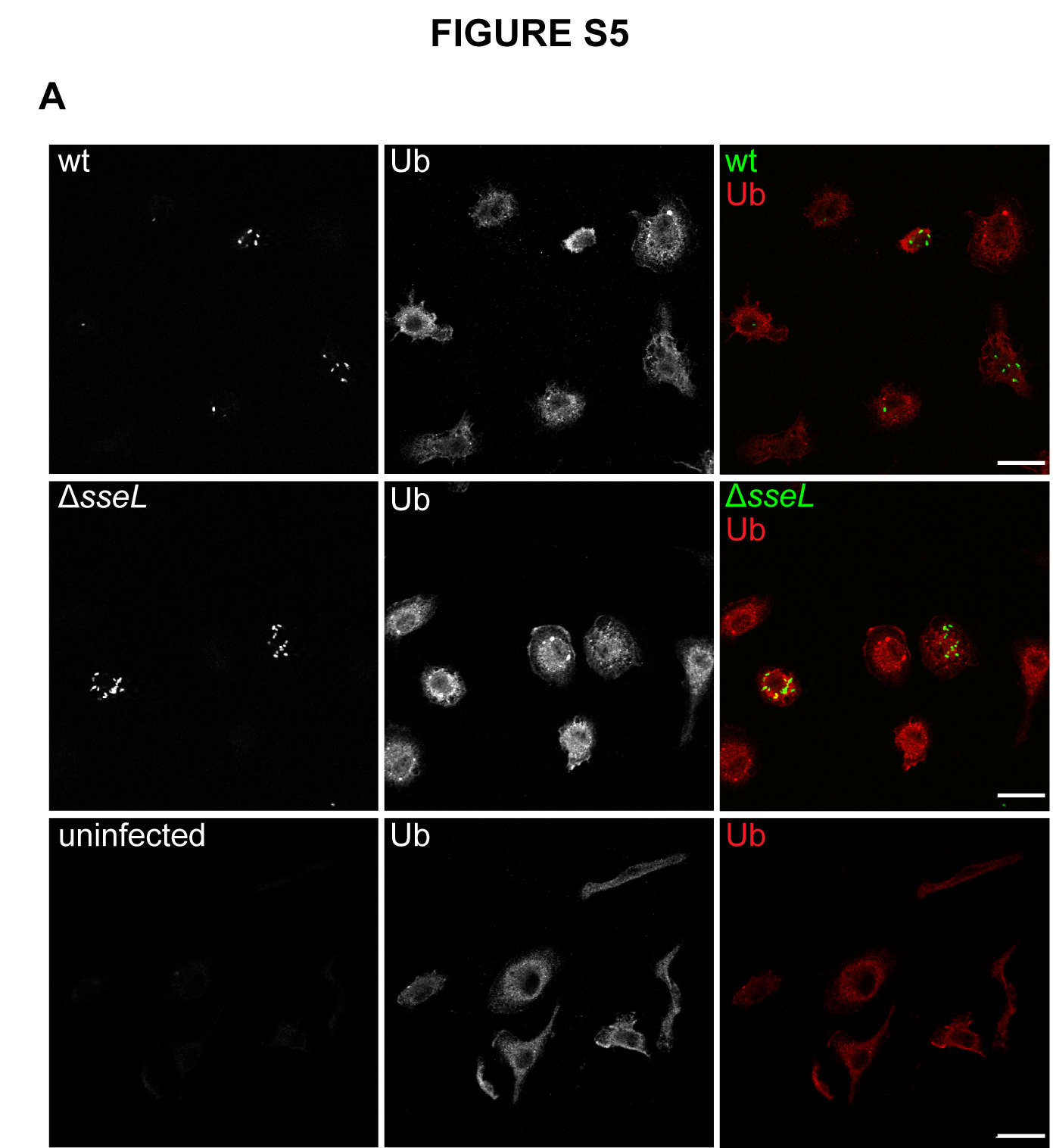

Supplement: Figure S5 — Confocal images of ubiquitin levels in primary macrophages. (A) Representative single confocal sections of murine primary bone marrow-derived macrophages infected with the indicated strains of GFP-expressing Salmonella (green) for 10 h or uninfected (and unexposed to bacterial products). Cells were fixed and labelled for ubiquitin (Ub, red) and analysed by confocal microscopy (scale bars, 20 µm). (TIF) [file ppat.1002743.s005.tif]
