# Supplementary material for: The Salmonella Deubiquitinase SseL Inhibits Selective Autophagy of Cytosolic Aggregates
Source: PLoS Pathog. 2012 Jun 14;8(6):e1002743. doi: 10.1371/journal.ppat.1002743 (PMC3375275; doi:10.1371/journal.ppat.1002743)
Supplement: Table S1 — Bacterial strains used in this work. (DOCX) [file ppat.1002743.s006.docx]

Table S1 – Strains used in this work.

| Name | Description | Source or reference |
| --- | --- | --- |
| 12023 | 12023 wild-type *S*. Typhimurium | NTCC |
| 12023 GFP | 12023 pFPV25.1 (Amp^R^) | NTCC |
| 12023 pDiGc | 12023 pDiGc (Amp^R^) | [[3](#_ENREF_3)] |
| Δ*ssaV* | Δ*ssaV* (Km^R^) in 12023 | [[60](#_ENREF_60)] |
| Δ*sseL::km* | Δ*sseL* (Km^R^) in 12023 | [[7](#_ENREF_7)] |
| Δ*sseL* | Δ*sseL* in 12023 | This study |
| Δ*sseL* GFP | Δ*sseL* pFPV25.1 (Amp^R^) | [[7](#_ENREF_7)] |
| Δ*sseL* pDiGc | Δ*sseL* pDiGc (Amp^R^) | This study |
| Δ*sseL,*pWSK29*sseL* | pWSK29*sseL*(HA)_2_ in Δ*sseL::km* (Km^R^, Amp^R^) | [[7](#_ENREF_7)] |
| Δ*sseL,pWSK29sseLc262a*-2HA | pWSK29*sseL*C262A(HA)_2_ in Δ*sseL* (Km^R^, Amp^R^) | [[7](#_ENREF_7)] |
| Δ*sseL,*pWSK129*sseL* pDiGc | pWSK29*sseL*(HA)_2_ and pDiGc in Δ*sseL* (Km^R^) | This study |
| Δ*sseL,*pWSK129*sseLc262a*-2HA pDiGc | pWSK29*sseL*C262A(HA)_2_ and pDiGc in Δ*sseL* (Km^R^) | [[7](#_ENREF_7)][[7](#_ENREF_7)] |
| Δ*avrA* | Δ*avrA* (Km^R^) in 12023 | Holden laboratory |
| Δ*sspH1* | Δ*sspH1* (Km^R^) in 12023 | Holden laboratory |
| Δ*sspH2* | Δ*sspH2* (Km^R^) in 12023 | Holden laboratory |
| Δ*slrP* | Δ*sspH2* (Km^R^) in 12023 | Andreas Baumler |
| Δ*sspH1*Δ*sspH2* | Δ*sspH2* (Km^R^) in Δ*sspH1* | This study |
| Δ*sspH1*Δ*sspH2* Δ*slrP* | Δ*slrP* (Km^R^) in Δ*sspH1*Δ*sspH2* | This study |
| *ΔsifAsseJ* | *ΔsseJ* in Δ*sifA*::mTn5 (Km^R^) | [[35](#_ENREF_35)] |
| *ΔsifAsopD2* | *ΔsopD2* (Km^R^) in Δ*sifA* | [[34](#_ENREF_34)] |
| *ΔsseJ* | *ΔsseJ* (Km^R^) in 12023 | [[35](#_ENREF_35)] |
| *ΔsseG* | *ΔsseG* (Km^R^) in 12023 | [[61](#_ENREF_61)] |
| *ΔsteC* | *ΔsteC* (Km^R^) in 12023 | [[38](#_ENREF_38)] |

Km^R^  = Kanamycin resistant; Amp^R^  = Ampicillin resistant
