# Supplementary material for: The Salmonella Deubiquitinase SseL Inhibits Selective Autophagy of Cytosolic Aggregates
Source: PLoS Pathog. 2012 Jun 14;8(6):e1002743. doi: 10.1371/journal.ppat.1002743 (PMC3375275; doi:10.1371/journal.ppat.1002743)
Supplement: Table S2 — Plasmids used in this work. (DOCX) [file ppat.1002743.s007.docx]

Table S2 – Plasmids used in this work.

| Plasmid | Description | Source or reference |
| --- | --- | --- |
| pFPV25.1 | rpsM::gfpmut3a promoter fusion in pFPV25 | [[68](#_ENREF_68)] |
| pDiGc | pDsRed.T3_S4T with *rpsM::gfpmut3a* into ClaI site | [[3](#_ENREF_3)] |
| pWSK29*sseL*-2HA | *sseL*(HA)_2_ in pWSK29 | [[7](#_ENREF_7)] |
| pWSK29*sseL*C262A-2HA | *sseL*C262A(HA)_2_ in pWSK29 | [[7](#_ENREF_7)] |
| pRK5::myc -*sseL* | myc-*sseL* in pRK5 | Holden laboratory |
| pWSK129*sseL*-2HA | *sseL*(HA)_2_ in pWSK129 | This study |
| pWSK129*sseL*C262A-2HA | *sseL*C262A(HA)_2_ in pWSK129 | This study |
| pGFPLC3 | GFP-LC3 in pEGFP | This study |
